# The relevance of cortisol co-secretion from aldosterone-producing adenomas

**DOI:** 10.1007/s42000-019-00114-8

**Published:** 2019-08-10

**Authors:** Padmanabh S. Bhatt, Amir H. Sam, Karim M. Meeran, Victoria Salem

**Affiliations:** 1 Division of Diabetes, Endocrinology and Metabolism, Department of Medicine, Imperial College London, 6th Floor Commonwealth Building, Hammersmith Hospital, Du Cane Road, London, W12 0NN UK; 2 Imperial College London NHS Trust, London, UK

**Keywords:** Adrenal cortex, Aldosterone, Cortisol, Hyperaldosteronism, Cushing’s syndrome, Co-secretion, Metabolic profile

## Abstract

**Aims and objectives:**

Adrenal adenomas are usually non-functioning, but can secrete aldosterone or cortisol. It has recently been suggested that many more adenomas than previously thought secrete more than one hormone. This has important implications for their clinical management. Our aim was to determine the frequency of cortisol co-secretion in primary hyperaldosteronism at our institution and investigate the difference in metabolic profiles and clinical outcomes between co-secreting and non-co-secreting patients.

**Design and patients:**

A retrospective study of 25 patients with primary hyperaldosteronism who also underwent formal dexamethasone suppression tests to determine cortisol co-secretion.

**Measurements:**

Post-dexamethasone suppression test cortisol, serum ALT, total cholesterol, HDL-cholesterol, LDL-cholesterol, HbA1C (were recorded) and mean arterial pressure are reported in this cohort of patients with primary hyperaldosteronism.

**Results:**

Four out of 25 patients with primary hyperaldosteronism failed dexamethasone suppression tests. This suggests a frequency of co-secretion ranging between 4 and 16%. No significant difference was found in serum ALT, total cholesterol, serum HDL-cholesterol, LDL-cholesterol and mean arterial blood pressure at presentation between co-secretors and non-co-secretors.

**Conclusion:**

A frequency range of 4–16% suggests that a significant proportion of patients with primary hyperaldosteronism co-secrete cortisol. Co-secretors did not have a worse metabolic profile than non-secretors. The impact of co-secretion on metabolic profile and surgical management remains unclear and warrants further study.

## Introduction

Primary hyperaldosteronism (PA) is an important, yet underdiagnosed cause of secondary hypertension, accounting for an estimated 10% of all cases of elevated blood pressure [1]. Adrenal corticosteroid autonomy (Cushing’s syndrome) is a rarer cause of hypertension, accounting for approximately 1% of cases, but associated with numerous other metabolic complications including weight gain and diabetes [2]. Aldosterone and cortisol co-secretion is a topic of debate, with recent studies demonstrating that it is possibly more common than previously understood. This is important, since removal of an undiagnosed cortisol-secreting adenoma (i.e. one that was thought to be aldosterone-producing only) could result in life-threatening cortisol deficiency postoperatively. Although large-scale studies on this topic are lacking, the prevalence of aldosterone and cortisol co-secretion is estimated to be between 5 and 21% [3, 4]. Even less is known about the clinical significance of this [5], and adrenal crisis after resecting a PA adenoma is very rarely reported.

In the setting of PA, early diagnosis and treatment of cortisol co-secretion are necessary not only to prevent hypoadrenalism post-operatively as mentioned but also to improve the dysmetabolic profile attributable to hypercortisolism and reduce the risk of other surgical complications, such as wound infections. Another important consideration is that cortisol co-secretion may invalidate the results of adrenal venous sampling, the gold-standard test for distinguishing unilateral from bilateral aldosterone hypersecretion and the decision basis for surgical versus medical management [4]. This is because an ipsilateral aldosterone and cortisol co-secreting adenoma may suppress contralateral cortisol secretion and thus give the impression of an improperly cannulated contralateral vein.

Our aim was to determine the frequency of cortisol and aldosterone co-secretion in PA patients at Imperial College London NHS Trust, which is a tertiary referral centre for adrenal tumours. Secondarily, we aimed to compare the metabolic profiles and clinical outcomes of co-secreting patients with their non-co-secreting counterparts.

## Materials and methods

### Data collection

This was a retrospective analysis of all patients undergoing adrenal vein sampling (AVS) for pre-surgical work-up (laterality) of biochemically confirmed primary hyperaldosteronism (PA) between 2011 and 2017 at Imperial College London NHS Trust. We identified those patients who also underwent formal dexamethasone suppression tests. The study was approved by the institutional audit committee. PA was diagnosed in patients in accordance with institutional guidelines, which are derived from Endocrine Society recommendations [6]. A raised plasma aldosterone/renin ratio is used as a screening test (> 80, where aldosterone is measured in pmol/l and plasma renin activity in pmol/L/min) and confirmed with a salt loading test, with two litres of 0.9% intravenous saline infused over 4 h; 4-h plasma aldosterone over 240 pmol/l is of the diagnostic cutoff for PA. Formal tests for cortisol hypersecretion were overnight dexamethasone suppression test (ODST) or low-dose dexamethasone suppression test (LDDST), with 9 am or 48-h serum cortisol levels > 50 nmol/l (1.81 μg/dl) suggesting adrenal autonomy.

Twenty-five patients were identified that had undergone AVS and formal dexamethasone suppression testing. Serum ALT (as a marker of liver steatosis and metabolic risk), cholesterol, HDL-cholesterol, LDL-cholesterol mean arterial blood pressure and glycaemic control at presentation were recorded, as indicators of metabolic profile. We also present brief case histories for these co-secretors.

Institutional guidelines were followed in determining adequate selectivity, lateralisation and contralateral suppression indices to quality control each procedure and to reliably subtype each patient. Adequate cannulation of the adrenal veins was confirmed by demonstrating a selectivity index of greater than two, compared with the IVC. Lateralisation index (LI) of two or greater with a contralateral suppression index (CSI) of under 0.5 was strongly suggestive of unilateral disease.

LI is the ratio of the aldosterone/cortisol ratio of the dominant side to the non-dominant side. CSI is the ratio of the aldosterone/cortisol ratio of the non-dominant side to the inferior vena cava.

### Assays

Aldosterone was assayed using a coat tube direct immunoassay (Siemens, UK). Plasma renin activity was assayed using an in-house enzyme kinetic assay (Imperial College NHS Trust Department of Clinical Biochemistry). Cortisol was measured using the Immulite 2000 assay (Siemens, UK).

### Statistics

Data was analysed by Prism (Version 7.0, GraphPad Software Inc., San Diego, CA, USA). D’Agostino and Pearson tests were used to assess normality of data distribution. All data is presented as median (interquartile range). Mann-Whitney tests were used to compare metabolic profiles between the two cohorts. In all cases, *p* < 0.05 was considered significant.

## Results

### Observed frequency

Four out of 25 patients with established PA were diagnosed as co-secretors after failing either or both of ODST or LDDST. In three of these cases, only one test for cortisol hypersecretion was performed, with room for discussion as to whether they truly were co-secretors (as detailed in the case studies). This suggests a co-secretion frequency of between 4 and 16%. The post-ODST range was 75 to 225 nM (2.71–8.16 μg/dl). Three patients underwent a unilateral adrenalectomy, of which two subsequently failed a short synacthen test, and a third patient had a 9 am cortisol of 20 nM (0.74 μg/dl). Table 1 describes biochemical characteristics and management that the co-secreting patients underwent.

**Table 1:** Biochemical characteristics and management of cortisol co-secreting PA patients

Patient ID	Aldosterone-renin ratio (ARR)	Aldosterone post-saline suppression test (pmol/l)	Cortisol post-overnight dexamethasone suppression test (nmol/l)	Cortisol post-low-dose dexamethasone suppression test (nmol/l)	Number of anti-hypertensive medications at presentation	Management
1	282	920	75	–	2	Unilateral adrenalectomy
2	> 574	1330	–	53	4	Unilateral adrenalectomy
3	> 721	490	225	61	2	Medical management
4	> 694	–	79	16	2	Unilateral adrenalectomy

### Metabolic profile

**Fig. 1:**
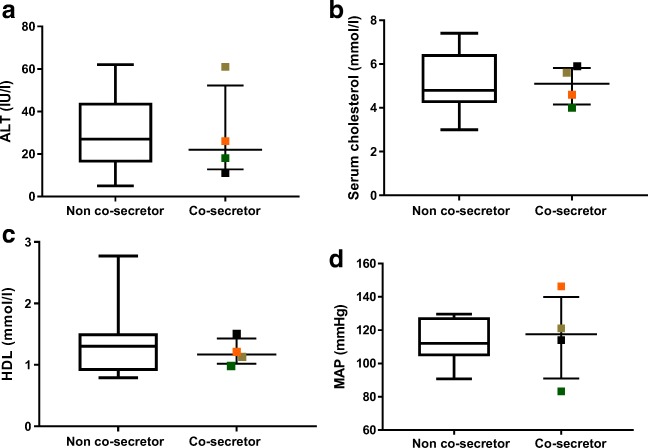
**a** Comparison of serum ALT at presentation between confirmed co-secretors and confirmed non-co-secretors. There was no significant difference in ALT at presentation between co-secretors (median 22 IU/l (52–12), range 11 to 61) and non-co-secretors (median 27 IU/l (44–16)). **b** Comparison of serum cholesterol at presentation between co-secretors and non-co-secretors. There was no significant difference in serum cholesterol at presentation between co-secretors (median 5.1 mM (5.8–4.3), range 4 to 5.9) and non-co-secretors (median 4.8 mM (6.5–4.2)). **c** Comparison of serum HDL at referral between co-secretors and non-co-secretors. There was no significant difference in serum HDL at presentation between co-secretors (median 1.2 mM (1.4–1.1), range 0.98 to 1.5) and non-co-secretors (median 1.3 mM (1.5–0.9)). **d** Comparison of mean arterial pressure at referral between co-secretors and non-co-secretors. There was no significant difference in MAP at presentation between co-secretors (median 118 mmHg (140–91), range 83 to 146) and non-co-secretors (median 112 mmHg (128–104)). Bars in the boxplots above indicate maximum and minimum values, whilst the box itself represents the interquartile range. Co-secretor data is shown in scatter plots, with median and interquartile range indicated by the bars

Serum ALT, cholesterol, HDL-cholesterol, LDL-cholesterol and mean arterial pressure were used as indictors of metabolic profile. No difference was observed in any of the above parameters (Fig. 1) between co-secreting patients and their non-co-secreting counterparts at presentation. The number of anti-hypertensive or potassium medications was also not different between the two groups (data not shown).

Post-treatment blood pressure was not readily available in the control group, but in the cases where it was available, no difference was seen between the co-secretor cohort and the control group (*U*-value 25, data not shown).

### AVS findings in co-secretors

Tables 2 and 3 illustrate the AVS raw results and indices from co-secretors, respectively. One dataset is missing due to technical difficulties cannulating the right adrenal vein.

**Table 2:** Comparison of co-secretors’ raw AVS results

Patient ID	CP	CL	CR	AP	AL	AR	Diagnosis
1	179	2094	*	740	1890	*	Right sided
2	448	2022	7888	1030	2310	50,000	Right sided
3	324	14,483	12,032	650	21,640	65,910	Bilateral
4	367	4057	10,954	460	1460	52,000	Right sided

*CP* peripheral cortisol, *CL* left adrenal vein cortisol, *CR* right adrenal vein cortisol, *AP* peripheral aldosterone, *AL* left adrenal vein aldosterone, *AR* right adrenal vein aldosterone

AVS of patient 1 was abandoned due to technical difficulties

Cortisol is measured in nmol/l; aldosterone is measured in pmol/l.

**Table 3:** Comparison of co-secretors’ AVS indices

Patient ID	CL/CP	CR/CP	A:CP	A:CL	A:CR	LI	CSI	Imaging results	Diagnosis
1	11.7	*	4.13	0.9	*	*	0.22	Right adrenal lesion	Left suppressed
2	4.51	17.61	2.30	1.14	6.34	5.55	0.50	2.7 cm right adrenal adenoma	Right sided
3	44.70	37.14	2.01	1.49	5.48	3.67	0.74	Left adrenal nodularity	Bilateral
4	11.05	29.85	1.25	0.36	4.75	13.19	0.29	3 cm right adrenal adenoma	Right sided

Lateralisation of aldosterone hypersecretion was diagnosed by a combination of AVS and radiological findings.

### Tumour size

Three of the co-secretors had unilateral adenomas on imaging. Their sizes were 1.9 cm, 2.7 cm and 3 cm.

The radiologists did not always report adenoma sizes in non-co-secretors, mostly due to inability to distinguish adenomas from hyperplasia, diffuse nodularity or sprawling and ill-defined morphology. The ones that were reported (*n* = 20) had a mean size of 1.4 cm.

## Brief case histories

### Case 1

Age at presentation, 63; gender, female; ethnicity, White British

Patient 1 was referred with a blood pressure of 110/70 mmHg on two anti-hypertensive agents. The patient was also diabetic and managed on metformin with HBA1c levels between 37 and 42 mmol/mol (5.5–6%) in the year prior to referral. However, due to her protracted history of diabetes and hypertension, patient 1 had already developed retinopathy and nephropathy. The patient also had a history of hypokalaemia and once required hospitalisation to normalise her serum potassium. On referral her HBA1c was 44 mmol/mol.

PA was diagnosed on the basis of a raised aldosterone-renin ratio and subsequent failure to suppress on saline infusion. Imaging suggested an obvious abnormality in the right adrenal gland. AVS was unsuccessful with the radiologist confirming failed cannulation of the right adrenal vein due to technical difficulties. The left adrenal was adequately cannulated and provided the following results:

- Left adrenal vein cortisol, 2094 nmol/l; left adrenal vein aldosterone, 1890 pmol/l
- IVC cortisol, 179 nmol/l
- IVC aldosterone, 740 pmol/l
- Left adrenal vein aldosterone/cortisol ratio, 0.90
- IVC aldosterone/cortisol ratio, 4.13
- Ratio of left adrenal vein aldosterone/cortisol ratio to IVC, 0.22

Thus, there is evidence of left adrenal suppression, suggesting a right adrenal focus of aldosterone hypersecretion.

The patient had no discriminatory features of Cushing’s syndrome, but had gained 10 kg over 7 months, and subsequently had interscapular fat pads. Patient 1 did not adequately suppress cortisol levels following an ODST with a cortisol of 75 nM (2.72 μg/dl). Her ACTH levels were suppressed at 10 ng/l, suggesting an adrenal focus of cortisol hypersecretion. Co-secretion was confirmed with a morning cortisol the day after surgery of 29 nM (1.05 μg/dl) and an inadequate response to a short tetracosactide test with a 1-h cortisol of 421 nM (15.3 μg/dl). Glucocorticoids were prescribed prior to discharge. The patient’s contralateral adrenal responded well and glucocorticoids were removed a few months later.

The patient’s blood pressure and serum potassium had normalised 18 months post-operatively, with a normal aldosterone-renin ratio. However, her HBA1c had slightly worsened to 49 mmol/mol. It is important to note in this case that clinical practice guidelines advocate the use of two tests for cortisol hypersecretion to confirm a diagnosis. In this case, only one ODST was performed (with an unequivocal result). However, post-operatively, there were two further pieces of evidence to suggest prior cortisol hypersecretion. Whilst neither post-operative morning cortisols nor post-operative tetracosactide tests have 100% specificity [7, 8], the accumulated data in this case led to a consensus diagnosis of co-secretion.

#### Case 2

Age at presentation, 57; gender, female; ethnicity, White British

Patient 2 was referred with a history of resistant hypertension and persistently low serum potassium. At referral, her blood pressure was 161/98 mmHg on four anti-hypertensive medications, and her serum potassium was 3.5 mM, without potassium supplements. Whilst awaiting definitive management, the patient had an episode of malignant hypertension with blood pressure of 240/120 mmHg, requiring a visit to the emergency department. This episode was associated with papilloedema and deteriorating renal function. The patient also had a history of aortic dissection and incomplete right bundle branch block. The patient’s mother was reported to have suffered from resistant hypertension and a fatal ruptured aortic aneurism. On referral her HBA1c was 38 mmol/mol.

A 2.7-cm right adrenal incidentaloma was discovered on routine imaging, and a subsequently raised aldosterone-renin ratio confirmed by saline suppression testing confirmed PA. Right lateralisation was confirmed by AVS.

Patient 2 did not have overt signs of Cushing’s syndrome and was non-diabetic. She did not suppress after a LDDST with a 48-h cortisol of 53 nM (1.92 μg/dl), and no further Cushing’s syndrome diagnostic tests were conducted. The patient had an ACTH level of 11.7 ng/l, suggesting an adrenal focus of cortisol hypersecretion. However, upon removal of the aldosterone-secreting tumour, the patient mounted an inadequate response to a post-operative short tetracosactide test with a 1-h cortisol of 320 nM (11.6 μg/dl). She was discharged on glucocorticoid replacement.

Three years post-operatively, the patient’s serum potassium had normalised, but she remained hypertensive on two anti-hypertensive agents. The patient remained in stage 3b chronic kidney disease and was still on glucocorticoid replacement, having failed attempts to be weaned off long tetracosactide testing. Her DHEAS level 4 years after AVS was under 0.4 μmol/l. Her HBA1c remained at 38 mmol/mol. Given the fact that this patient only failed one diagnostic test for hypercortisolism (guidelines suggest a second test improves specificity) and only one post-operative test for adrenal suppression (which has also been shown to be imperfect in this setting [8]), we report this case of co-secretion with caution, and this adds to the lower range in our reported frequency.

#### Case 3

Age at presentation, 30; gender, female; ethnicity, Black African

Patient 3 suffered from post-partum hypertension and was referred with a blood pressure of 191/124 mmHg and serum potassium of 2.7 mM on two anti-hypertensive medications and 1.9 g of potassium supplements daily. The patient subsequently developed hypertensive retinopathy. It was recommended that she stop breastfeeding in order to start broader medication for blood pressure optimisation. On referral, the patient had a fasting plasma glucose concentration of 4 mmol/L (72 mg/dl).

A raised aldosterone-renin ratio suggested PA. Patient 3 was deemed to have bilateral disease based on discordant AVS and imaging findings: although she had a convincing LI of 3.6 suggesting right sided disease, the CSI was not completely suppressed at 0.75. Moreover, imaging suggested significant nodularity of the left adrenal gland. Medical therapy was deemed suitable in light of the conflicting lateralisation data and patient preference.

Although not overtly Cushingoid, the patient did not suppress cortisol production with two LDDST with 48-h cortisol measurements of 61 nmol/l (2.21 μg/dl) and 53 nmol/l (1.92 μg/dl). The patient also did not suppress cortisol secretion on further tests including an overnight dexamethasone suppression test with a morning cortisol of 225 nmol (8.16 μg/dl) and a high-dose dexamethasone suppression with a 48 h cortisol of 59 nmol/l (2.13 μg/dl). Her serum potassium concentration remains at the lower end of normal at 3.7 mM on 1.9 g of potassium supplements and blood pressure was inadequately controlled at 149/89 mmHg on two anti-hypertensive agents. She did not attend follow-up for further discussion about management options.

#### Case 4

Age at presentation, 52; gender, male; ethnicity, Black African

Patient 4 was referred with a 10-year history of hypertension and hypokalaemia. On referral, blood pressure was 154/94 mmHg on two anti-hypertensive agents and serum potassium was 2.9 mM. Although the patient himself was non-diabetic with an HBA1c of 34 mmol/mol, he had a dyslipidaemic profile with serum total cholesterol and LDL-cholesterol raised at 5.9 mM (228 mg/dl) and 3.73 mM (144 mg/dl), respectively.

PA was suggested by a raised aldosterone-renin ratio and lateralised to the right adrenal gland based on AVS and imaging. Although the patient did not display discriminatory Cushingoid features, he did not adequately suppress cortisol secretion after an ODST with morning cortisol of 79 nM (2.86 μg/dl). However, it was subsequently noted that he was a shift worker, and shift work is understood to disturb the normal circadian release of cortisol. Subsequently the patient adequately suppressed his cortisol after an LDDST; multiple 24-h UFCs were normal and a repeat ODST demonstrated a 9 am cortisol of 16 nM (0.58 μg/dl). Thus, cortisol co-secretion seemed unlikely. His ACTH level was 16.4 ng/l. The patient was to undergo a right retroperitoneoscopic adrenalectomy but it had to be converted to an open, laparatomic approach due to adherence of the adrenal gland to the liver capsule. On day one post-operatively, patient 4 had a morning cortisol of below 20 nM (0.73 μg/dl). Given the preceding concern about the possibility of co-secretion in this patient, a decision was made to err on the side of caution, and he was discharged on glucocorticoid replacement therapy. Three months post-operatively, patient 4 mounted an adequate response after a short tetracosactide test, and corticosteroid supplementation was stopped.

The patient recovered well and 4 years after presentation is normokalaemic and normotensive with BP 140/80 mmHg on one anti-hypertensive agent. We have chosen to include this patient in this cohort of case reports again to highlight the nuanced clinical decision-making that is often required in these scenarios.

## Discussion

We report a frequency range of 4–16% for cortisol co-secretion (as diagnosed on ODST or LDDST) in patients with PA. It is important to note here that there could have existed some bias in this figure, since we do not routinely test all our patients for co-secretion, and therefore only those where the clinician had a suspicion of co-secretion would have been tested for it. Therefore, we have refrained from the use of the word prevalence. Nevertheless, our range of 4–16% reflects the reported prevalence of this phenomenon in other studies [3, 9, 10]. Two of these patients had evidence of contralateral gland suppression (i.e. risk of adrenal crisis) on post-operative tetracosactide testing after adrenalectomy.

On the other hand, actual incidents of life-threatening adrenal crisis after surgical treatment for PA are extremely uncommon. Recently, Arlt et al. reported that PA patients have 25% higher total urinary glucocorticoid excretion compared with controls (in the same range as patients with confirmed adrenal corticosteroid autonomy) [5]. In that study, surrogate risk markers such as BMI and insulin resistance positively correlated with the degree of urinary glucocorticoid excretion. Interestingly, the same group noted that far fewer PA patients inadequately suppressed after formal Cushing’s syndrome confirmatory tests, despite elevated urinary glucocorticoids [5]. There is some evidence that patients without cortisol hypersecretion pre-operatively may display biochemical features of adrenal insufficiency in the immediate post-operative period [7, 8].

Our findings suggest that co-secretion of aldosterone and cortisol may be more common than previously appreciated but that the clinical significance of this remains unclear. Furthermore, given the apparent range in the degree and timing of co-secretion, suppression tests may be inadequate in assessing this phenomenon.

There is evidence that aldosterone and cortisol co-secreting adenomas can confound AVS. Adrenal vein cortisol concentrations are measured to determine adequate cannulation. The aldosterone to cortisol ratio is compared between the two veins, which accounts for dilution effects of the inferior phrenic vein on the left and the hepatic vein on the right. An ipsilateral aldosterone and cortisol co-secreting adenoma may suppress contralateral cortisol secretion and thus give the impression of an improperly cannulated contralateral vein [11, 12]. Indeed, a study discovered that six out of eight patients with adrenal Cushing’s syndrome had sufficiently suppressed contralateral adrenal vein cortisol to indicate failed cannulation, though aldosterone levels confirmed adequate cannulation [4]. Although Goupil et al. studied patients with Cushing’s syndrome without primary hyperaldosteronism, aldosterone and cortisol co-secreting adenoma findings may be similar. Additionally, cortisol co-secreting adenomas may suppress the ipsilateral aldosterone-cortisol ratio while enhancing the contralateral aldosterone-cortisol ratio due to cortisol suppression. This picture may falsely indicate bilateral aldosterone hypersecretion, because the ipsilateral to contralateral aldosterone-cortisol ratio may not suppress adequately. In the small series of patients described here, one AVS was technically challenging and abandoned. There were no cases of “failed” AVS based on the possible spurious biochemical diagnosis of an inadequately cannulated adrenal vein. Our previously published data [13] suggests that our AVS “failure” rate is < 10%—far smaller than the possible prevalence of bi-secretion. In conclusion, cortisol co-secreting adenomas may potentially deprive patients of adrenalectomy. Perhaps the use of adrenal vein metanephrines instead of cortisol in co-secreting patients may be a more robust tool [14].

Current clinical consensus is that adrenal autonomy leading to hypercortisolism worsens metabolic profiles [2]. However, there was no significant difference between the metabolic profiles of co-secretors and non-co-secretors in our cohort. This unexpected finding may be due to the small numbers of patients identified with this rare co-secreting abnormality (four co-secretors were identified, compared with 21 PA patients with negative dexamethasone suppression tests). Nevertheless, it is also worth mentioning that this finding corroborates other similar small case series [3]. It remains uncertain to what extent the lack of metabolic complications from cortisol co-secretion in PA patients is a true phenomenon related to a low/clinically insignificant secreted cortisol burden is or because these patients are diagnosed much earlier.

It might be theorised that all aldosterone-secreting adenomas co-secrete cortisol to a certain extent, but clinical hypercortisolism is only apparent in large tumours [15]. Funder et al. did indeed report that aldosterone and cortisol co-secreting adenomas are usually larger than PA adenomas [6]. Furthermore, histologically these tumours are probably more heterogeneous than originally described, with aldosteronomas rarely consisting of purely zona glomerulosa-like cells [16]. We were not able to corroborate any of these findings in this study.

This study drew on retrospective data, and we have highlighted in the case histories where certain data may not have been available and how low numbers in our co-secretor cohort will have affected the ability to draw firm conclusions. Furthermore, the nuances involved in interpreting multiple suppression tests, each of which does not have perfect sensitivity or specificity, add to the clinical endocrinologist’s uncertainty. For example, evidence that immediate post-operative morning cortisol and short tetracosactide tests might falsely predict hypoadrenalism might mean some patients may not have had clinically significant co-secretion. Clearly, a larger prospective study is required, which is being conducted at our institution. This study will incorporate unbiased prospective endocrine testing for both conditions at presentation of either, with full follow-up including histological examination of any tumours that are removed. There is evidence that immunostaining for enzymes such as 3βHSD, CYP11B1, CYP11B2 and CYP21A2 is informative in delineating precisely which hormones are hypersecreted [5]; unfortunately this was outside the scope of this study.

Further work is required to understand how best to manage PA patients and the clinical relevance of cortisol co-secretion. However, based on this study, we have changed our clinical practice to now formally test all our patients with PA for cortisol excess.
